# Tasco^®^, a Product of *Ascophyllum nodosum*, Imparts Thermal Stress Tolerance in *Caenorhabditis elegans*

**DOI:** 10.3390/md9112256

**Published:** 2011-11-08

**Authors:** Saveetha Kandasamy, Di Fan, Jatinder Singh Sangha, Wajahatullah Khan, Franklin Evans, Alan T. Critchley, Balakrishnan Prithiviraj

**Affiliations:** 1 Department of Environmental Sciences, Nova Scotia Agricultural College, P.O. Box 550, Truro NS B2B 5E3, Canada; E-Mails: skandasamy@nsac.ca (S.K.); fand@nsac.ca (D.F.); jsangha@nsac.ca (J.S.S.); 2 Acadian Seaplants Limited, 30 Brown Avenue, Dartmouth NS B3B 1X8, Canada; E-Mails: fevans@acadian.ca (F.E.); alan.critchley@acadian.ca (A.T.C.); 3 Genome Research Chair Unit, Department of Biochemistry, College of Science, King Saud University, PO Box 2455, Riyadh 11451, Saudi Arabia; E-Mail: wkhan@ksu.edu.sa

**Keywords:** Tasco^®^, *Ascophyllum nodosum*, *Caenorhabiditis elegans*, longevity, thermo tolerance, stress, proteomics, gene expression

## Abstract

Tasco^®^, a commercial product manufactured from the brown alga *Ascophyllum nodosum*, has been shown to impart thermal stress tolerance in animals. We investigated the physiological, biochemical and molecular bases of this induced thermal stress tolerance using the invertebrate animal model, *Caenorhabiditis elegans*. Tasco^®^ water extract (TWE) at 300 μg/mL significantly enhanced thermal stress tolerance as well as extended the life span of *C. elegans*. The mean survival rate of the model animals under thermal stress (35 °C) treated with 300 μg/mL and 600 μg/mL TWE, respectively, was 68% and 71% higher than the control animals. However, the TWE treatments did not affect the nematode body length, fertility or the cellular localization of *daf-16*. On the contrary, TWE under thermal stress significantly increased the pharyngeal pumping rate in treated animals compared to the control. Treatment with TWE also showed differential protein expression profiles over control following 2D gel-electrophoresis analysis. Furthermore, TWE significantly altered the expression of at least 40 proteins under thermal stress; among these proteins 34 were up-regulated while six were down-regulated. Mass spectroscopy analysis of the proteins altered by TWE treatment revealed that these proteins were related to heat stress tolerance, energy metabolism and a muscle structure related protein. Among them heat shock proteins, superoxide dismutase, glutathione peroxidase, aldehyde dehydrogenase, saposin-like proteins 20, myosin regulatory light chain 1, cytochrome c oxidase RAS-like, GTP-binding protein RHO A, OS were significantly up-regulated, while eukaryotic translation initiation factor 5A-1 OS, 60S ribosomal protein L18 OS, peroxiredoxin protein 2 were down regulated by TWE treatment. These results were further validated by gene expression and reporter gene expression analyses. Overall results indicate that the water soluble components of Tasco^®^ imparted thermal stress tolerance in the *C. elegans* by altering stress related biochemical pathways.

## 1. Introduction

The ocean, makes up more than 70% of the earth’s surface, contains about 90% of the world’s living biomass, and marine species contribute to almost half of the total worldwide biodiversity [1]. Recently, there has been an increased consumer interest concerning the significance of diet on health and wellness. Consequently, the search for natural products that have the potential to improve health and quality of life has intensified [2]. Apart from an important source of nutrition, marine organisms are also rich in functionally active chemicals. The brown algae (Phaeophyta), *Ascophyllum nodosum* L. Le Jolis, dominates the rocky intertidal shores of Atlantic Canada and Northern Europe [3–5]. Tasco^®^, a commercial product prepared from the brown alga *Ascophyllum nodosum*, is a natural animal feed ingredient that has shown positive effects as an antioxidant and improved stress tolerance and immune function [6]. The product is recognized as safe (GRAS) by the Association of American Feed Control Officials (AAFCO) [7]. Tasco^®^ offers animal producers an effective means to improve health by promoting improved status of the gastro-intestinal (GI) tract microflora, increasing stress resistance of the animal through activation of the immune system and addressing sub-clinical infection in a manner different from that of conventional antibiotics [8]. It has also been reported to lower core body temperatures of cattle in hot weather as well as stimulating higher core body temperatures in colder weather [6,9]. However, the chemical components in Tasco^®^ eliciting these beneficial responses or the biochemical and molecular basis of these effects, are not known.

The soil nematode *C. elegans* is an attractive model to study the genetics and biochemistry of the animal endocrine system, and can provide insight into signaling pathways relevant to human and higher animal systems. The model invertebrate has been used to characterize mechanisms related to stress responses in animals [10,11]. The physiological and biochemical mechanisms of stress tolerance in *C. elegans* have extensively been studied and there is considerable overlap of signaling pathways between the model nematode and higher animals. In this study, we used *C. elegans* to investigate the mechanism(s) of Tasco^®^-imparted thermal stress tolerance combining physiological, biochemical, molecular and proteomic approaches.

## 2. Results

### 2.1. Tasco^®^ Water Extract (TWE)-Treated Nematodes Exhibited Enhanced Thermal Tolerance

To investigate the protective effect of TWE against heat stress in *C. elegans* wild type N2, the treated nematodes were exposed to higher temperature (30 or 35 °C) and survival rate was observed. TWE at a concentration of 300 μg/mL showed the largest positive effect and enhanced thermo tolerance in the worm at both temperatures (data not shown). When the adult worms were exposed to mild heat stress (30 °C), about 60% enhancement in mean life span was observed in TWE-treatment. Similarly, TWE treatment also had a positive effect (*P* < 0.05) on survival rates of worms at higher temperature (35 °C). At the time when all of the control worms died, 48% of the nematodes treated with TWE (300 μg/mL) were alive. The mean survival rate was significantly increased (68%) by 300 μg/mL TWE treatment compared to the control.

### 2.2. TWE-Treatment Does Not Affect Cellular Localization of *daf-16* in *C. elegans*

**Figure 1:**
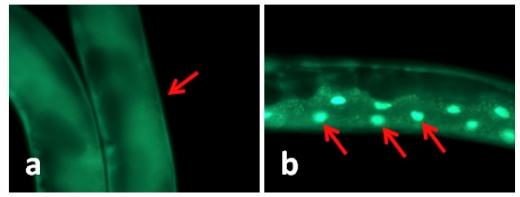

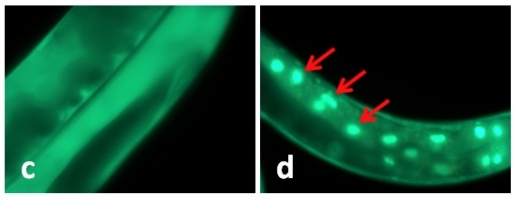
Water extract of Tasco^®^ (TWE) does not affect cellular translocation of *daf-16::gfp* in transgenic *C. elegans* (TJ356). (**a**) Control (20 °C) *daf-16* cytosolic localization; (**b**) *daf-16* nuclear translocation (worm kept at 35 °C for 2 h, then at 20 °C for 4 h); (**c**) *daf-16* cytosolic localization following TWE 300 μg/mL treatment at 20 °C; (**d**) *daf-16* nuclear translocation after TWE 300 μg/mL treatment at 35 °C for 2 h followed by 4 h at 20 °C (Intestinal part magnification 20×).

In *C. elegans* the transcription factor *daf-16* mediates the expression of a number of genes that leads to stress tolerance and improved longevity. To study whether *daf-16* was involved in TWE-elicited thermal tolerance, the effect of TWE on sub-cellular localization of *daf-16* was examined in a transgenic nematode strain TJ356. In this strain, *daf-16* is fused to a green fluorescent protein (GFP) making it possible to observe sub-cellular localization of *daf-16* in the living worm. Under normal growth conditions, *daf-16* is localized primarily in the cytosol, but under heat stress it is rapidly trans located into the nucleus [12]. Our results clearly showed that the *daf-16::gfp* protein was trans-located into the nucleus of the intestinal cells under heat stress (Figure 1). TWE treatment did not affect nuclear translocation of *daf-16::gfp*. The translocation of the fusion protein was similar in both the control and TWE treated worm (Figure 1).

**Figure 2:**
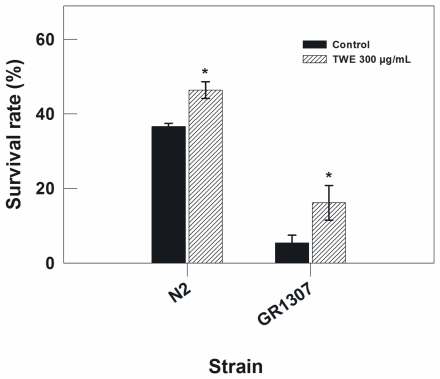
Thermal tolerance in N2 and *daf-16 C. elegans* (GR1307) treated with or without TWE, from egg stage up to 3 days (* *P* < 0.05 *vs.* control). N2 worms were exposed to 35 °C for 8 h, and *daf-16* mutants for 7 h.

To further rule out the possible involvement of *daf-16* pathway in the TWE-induced thermal stress tolerance, the response of the wild type strain (Bristol N2) was compared to a *daf-16* mutant (mgDf50). The wild type and the *daf-16* mutant (mgDf50) nematode were exposed to heat stress (35 °C). The result showed that TWE treatment enhanced the thermal tolerance of both the wild type and the *daf-16* mutant with a mean increase of 1.3 and 2.2-fold, respectively (Figure 2). This indicates that the effect of TWE on heat tolerance in *C. elegans* is independent of *daf-16*.

### 2.3. TWE Treatment Prolongs Life Span in Wild Type *C. elegans*

**Figure 3:**
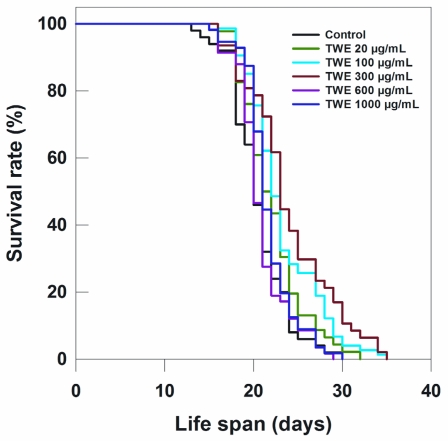
Effects of TWE on longevity of *C. elegans* N2 started from egg stage at 20 °C.

To determine the effect of TWE on lifespan of *C. elegans* N2 strain, the worms were treated with different concentrations of TWE. Low concentration (20 μg/mL) of TWE had no effect on longevity (Table 1; Figure 3). However, the higher concentrations (100 and 300 μg/mL) significantly extended the mean life span by 2 days or more. On the other hand, higher concentrations (e.g., 600 and 1000 μg/mL) had no significant effect on the life span of *C. elegans* as compared to the 300 μg/mL (Figure 3; Table 1).

**Figure 4:**
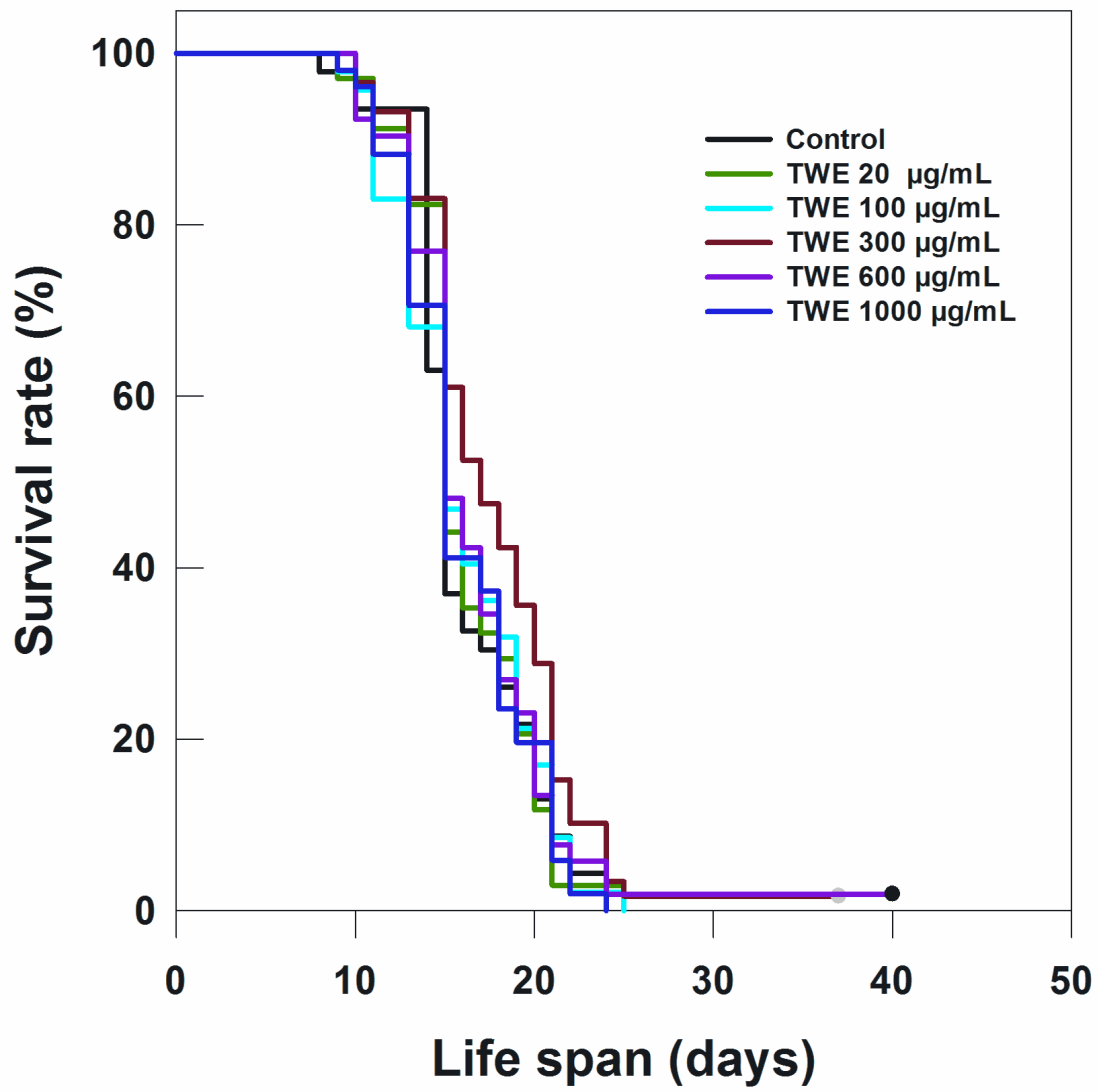
Effect of TWE on longevity of *C. elegans* N2 when treated at 6 days after hatching at 20 °C.

The most effective concentration TWE 300 μg/mL not only extended the mean life span of *C. elegans* by 3 days (indicative of an extension of mean life span by 17%), but also increased the maximum life span by approximately 17% (5 days) (data not shown). Because TWE treatments were present throughout the entire life stages of *C. elegans* (starting from the egg stage and onward), the question arose whether the TWE extended life span when the nematode was exposed at a later life stage. Interestingly, the median concentration TWE (300 μg/mL) extended the mean life span (17.76 days) (*P* < 0.05) compared to the control (16.13 days) (Figure 4).

### 2.4. Effect of TWE Treatment on Body Size, Pharyngeal Pumping Rate, and Fecundity in *C. elegans*

TWE treatment did not affect body length of nematodes. Average body length was 1.23 mm in TWE-treated worms compared to 1.30 mm in the control (data not shown). Similarly, the TWE treatment had no effect on body width. The average width was 66.0 μm in controls and 65.6 μm in the TWE-treated worms (data not shown).

**Figure 5:**
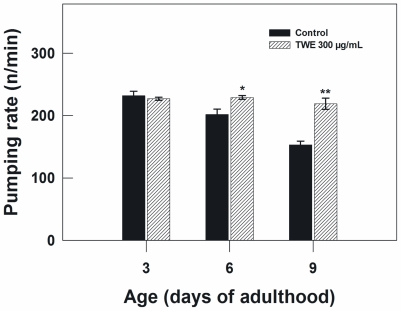
Effect of TWE 300 μg/mL and control on the pharyngeal pumping rate in *C. elegans* (treated at young adult stage). The treatments marked with asterisks * and ** were significantly different (*P* < 0.05 and *P* < 0.01, respectively; Tukeys HSD test) from the control.

The pharyngeal pumping rate in TWE-treated worms and controls were measured to examine the possibility that TWE treatment may have perhaps reduced the food intake of *C. elegans*, which in turn may lead to caloric restriction and as a result increased life span. *C. elegans* uses the pharynx to pump in the bacteria upon which it feeds and transports them to the intestine. The speed of pharyngeal contraction declines with age and the reduction in pumping rate has been applied as one of the indices of the decline of overall physiological functions. The age-dependent decline in pumping rate from day 3–9 of adulthood was apparent in the control. However, the control nematodes exhibited a significant decline in pharyngeal pumping rate as compared to TWE-treated on day 6 and 9 of adulthood (Figure 5).

**Figure 6:**
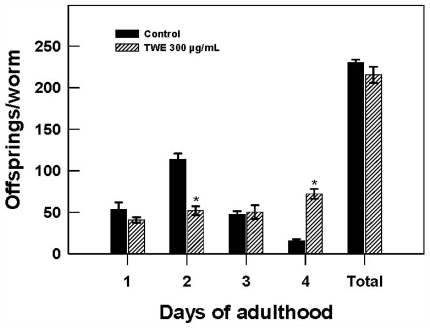
Effect of TWE 300 μg/mL and control on changes in reproductive output of *C. elegans* N2 during exposure to TWE. The treatments marked with asterisks * is significantly different (*P* < 0.05; Tukeys HSD test) from the control.

To test the effect of TWE treatment on *C. elegans* reproduction, the distribution of daily reproductive output and overall brood size were recorded. Reproduction was observed on day 1 and lasted for 4 days of adulthood. No significant differences was found in the brood size, averaging 230 (control) and 215 (TWE-treated) per worm. However, it was interesting to note that TWE-treated nematodes showed a significant delay in fecundity with a >2 fold decrease on day 2, but showed a surprising >4 fold increase in fecundity on day 4 (Figure 6).

### 2.5. TWE Affects the Expression of a Number of Stress Related Genes in *C. elegans*

**Figure 7:**
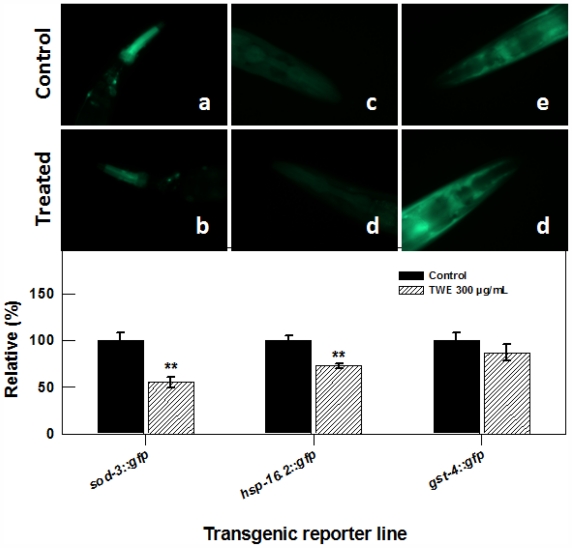
Effect of TWE (300 μg/mL) on the expression of stress response genes in *C. elegans* (**a**) the *sod-3::gfp C. elegans* (control); (**b**) the *sod-3::gfp C. elegans* following TWE treatment; (**c**) the *hsp-16.2::gfp C. elegans* (control); (**d**) the *hsp-16.2::gfp C. elegans* following TWE treatment; (**e**) the *gst-4::gfp C. elegans* (control); (**f**) the *gst-4::gfp C. elegans* following TWE treatment. The treatments marked with asterisks ** is significantly different (*P* < 0.01; Tukeys HSD test) from the control.

To elucidate whether TWE treatment elicits stress related molecular responses in *C. elegans*, we studied transgenic lines of superoxide dismutases (*sod-3*), antioxidant enzymes also important regulators of life span and stress resistance, stress-responsive glutathione S-transferase (*gst-4*) that confers resistance to oxidative stress and *hsp-16.2.* Transgenic strains of nematode *sod-3::gfp* (*cf1553*), *gst-4::gfp* (*cl2166*) and *hsp-16.2::gfp* (*cl2070*) were treated with TWE and exposed to thermal stress. The expression of *sod3*, *gst4* and *hsp16.2* was examined by observing the intensity of green fluorescent protein. TWE treatment significantly reduced the fluorescence in nematode carrying the *sod-3::gfp* (60% less compared to the control, Figure 7), indicating that TWE-treatment did not increase the expression of the *sod-3* gene. Similarly, TWE treatment significantly reduced the fluorescence in the *hsp-16.2::gfp* strain after a 2 h heat-shock (at 35 °C), followed by 4 h recovery at 20 °C. TWE (300 μg/mL) concentration reduced fluorescence in *C. elegans* strain *gst-4::gfp* as compared to untreated control (Figure 7).

### 2.6. Measurement of Reactive Oxygen Species (ROS)

**Figure 8:**
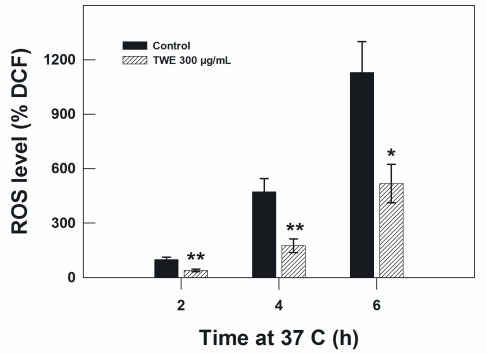
Effect of TWE (300 μg/mL) on ROS accumulation in *C. elegans* N2 strain. Results are expressed as DCF (2,7-dichlorofluorescein diacetate) relative to the control.

Because the heat stress effect and aging is related to oxidative stress, we measured the concentration of intracellular ROS in *C. elegans*. In the TWE-treated wild type N2 worms, TWE treatment significantly reduced the concentration of ROS under oxidative stress (300 μM juglone) compared to worms without TWE at 2 h, 4 h and 6 h of treatment (Figure 8).

### 2.7. Effect of TWE Treatment on the Expression of Stress Related Genes in *C. elegans*

**Figure 9:**
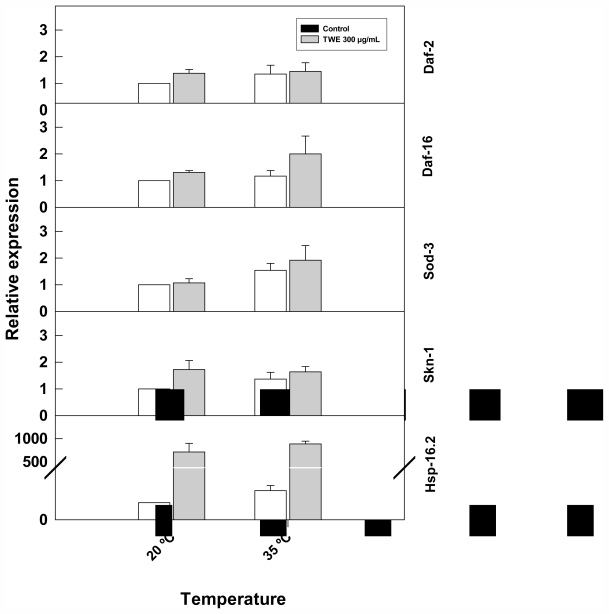
Effect of TWE (300 μg/mL) on the expression of stress response genes in *C. elegans*. Eggs of *C. elegans* strain N2 eggs were transferred to control or TWE-supplemented NGM plates until the young adulthood. The worms were then heat-shocked (35 °C) for 2 h. The relative gene expression of *hsp-16.2*, *daf-16*, *sod-3*, and *skn-1* were determined using quantitative real-time PCR. Transcript abundance of each selected gene is expressed relative to the expression in control nematode using the 2^−ΔΔCT^ method. Error bars represent SE of the mean of three independent runs.

TWE treatment altered the expression of stress-related genes (*hsp-16.2*, and *skn-1*) (Figure 9). The expression of *hsp-16.2*, increased considerably with heat stress and was higher in TWE-treated worms compared to the control. Similarly, skn-1 was up- regulated in TWE treatment. In contrast, the expression of *daf-2*, *sod-3*, *and daf-16* genes remained unchanged under heat stress in both the TWE treatment and the control.

### 2.8. Effect of TWE on *C. elegans* Proteome under Thermal Stress

**Figure 10:**
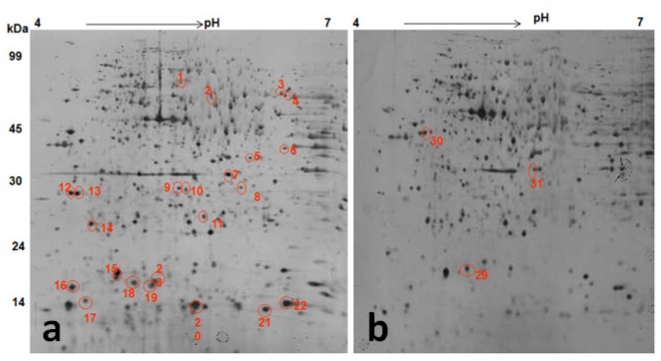
2DE gel pattern of protein extracted from TWE treated stressed (**A**) and control stressed (**B**) *C. elegans* at pH 4–7, non linear IPG strip, followed by a 12% SDS–polyacrylamide gel. The gel was stained with silver. Identified differentially up-regulated (**A**) and down-regulated (**B**) protein spots were circled and numbered in red color.

Proteomic changes induced by TWE treatment under thermal stress was studied by 2D gel electrophoresis analysis of the total protein (Figure 10). Most *C. elegans* proteins resolved at pH 4–7 and about 1000 spots were detected on a SDS-PAGE. Protein spots of TWE-treated and control worms were compared under thermal stress and non-stressed conditions. Differentially expressed protein spots were then isolated, digested with trypsin, treated with POROS R2 and R3 resins, and analyzed by MALDI-TOF-MS or MALDI-TOF-MS/MS. The location of the corresponding peak for each protein and its mass and pI were confirmed (Table 2). Analysis of *C. elegans* total proteome under heat stress and under non-stressed conditions revealed 40 differentially expressed proteins. Among the 40 proteins, 34 proteins were up-regulated and 6 were down-regulated (Table 2). The TWE specific differentially expressed proteins of *C. elegans* were functionally characterized and listed in Table 2.

### 2.9. Functional Prediction of Hypothetical Proteins

To study the probable function of the identified proteins, a standard protein BLAST search was performed using NCBI protein database [13] (Table 2) according to the Worm pep database. Interestingly, of the 40 proteins identified in this study, more than 80% of the proteins were “hypothetical proteins”. Analysis of PMF data of 40 proteins derived by MS analysis using the MASCOT search algorithm showed functional homology to the following proteins: CAEEL Putative uncharacterized protein; putative aldehyde dehydrogenase family 7 member A1 homolog; heat shock protein Hsp-16.11/16.48/16.49/25; 40S ribosomal protein SA OS; saposin-like protein family protein 20; carbamoyl-phosphate synthase L chain ATP-binding protein OS; superoxide dismutase OS; sensory axon guidance protein 7; myosin regulatory light chain 1 OS; cytochrome c oxidase subunit 5A; ATP synthase subunit beta, mitochondrial OS; glutathione peroxidase R05H10.5 OS; galectin OS; 60S ribosomal protein L18 OS; and peroxiredoxin protein 2. Based on descriptions derived from three separate databases (GOA, KEGG, NCBI KOG), all the identified proteins were classified into nine functional classes: (1) general metabolic process, (2) energy metabolism, (3) nucleotide/Protein binding, (4) electron transport, (5) protein synthesis, (6) proteolysis, (7) response to heat, (8) stress response, and (9) signal transduction.

#### 2.9.1. Signal Transduction

**Figure 11:**
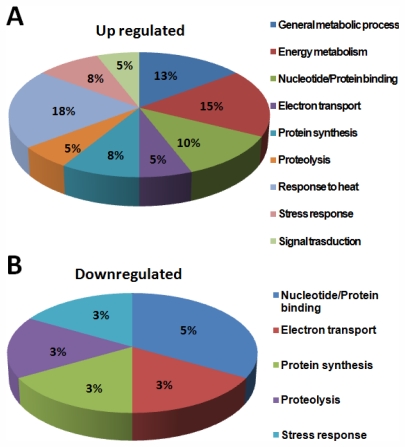
Functional distribution of identified proteins involved in different metabolic processes.

The category “general metabolism” accounts for about 40% of the total identified proteins. About 80% of proteins in this class were enzymes, involved in the metabolic processing of amino acids, carbohydrates, lipids, nucleotides and co-factors (Figure 11).

#### 2.9.2. Temperature Specific Marker Proteins

Comparison of *C. elegans* protein spots under heat stressed (at 35 °C) and normal (at 20 °C) condition showed differential expression only in stressed nematodes treated with TWE. It was observed that three heat shock proteins were over-expressed following TWE treatment. Several metabolic enzymes (Table 2) were also over-expressed exclusively in TWE-treated nematodes. A heat shock protein 17 and a 25 kDa protein (spot 4) were characteristically high in abundance in TWE-treated worms.

The HSP multi-gene family of *C. elegans* has at-least nine members [14] and although in this study a number of identified proteins were directly or indirectly related to heat stress metabolism, four of them were identified in TWE-treated *C. elegans* under heat stress (35 °C) (HSP11, HSP16.1, HSP16.48, and HSP25) from the protein spots marked as number 8, 18, 22, and 23, respectively. Interestingly, three potential metabolic proteins namely carbamoyl-phosphate synthase, cytochrome c oxidase and ATP synthase and three stress related defensive proteins viz., aldehyde dehydrogenase, superoxide dismutase and probable glutathione peroxidase were also specifically over expressed in TWE-treated worms.

## 3. Discussion

Despite the common use of *Ascophyllum nodosum* supplements such as Tasco^®^ in animal diets that improve stress tolerance [15,16], the precise mechanisms associated with such beneficial effects are not fully understood. This study used the nematode, *C. elegans* as a model to elucidate the mode of action of an *A. nodosum* commercial product Tasco^®^. The free-living soil nematode *C. elegans* has been a useful model system in ageing and drug screening research [17,18] and has previously been used to understand heat stress tolerance mechanisms [19]. The mechanistic findings with this model are directly relevant to higher animals because it shares a number of conserved biochemical pathways with mammals [20,21]. High temperature is a major stress in animals and a range of mechanisms are involved to combat such assault [22–24]. The present research demonstrated that exposure of *C. elegans* to water extract of Tasco^®^ significantly improved thermal tolerance and reduced the oxidative stress.

TWE treatment extended the life span of wild type N2 strain of *C. elegans* under heat stress. Optimal effect was observed in TWE 300 μg/mL concentration. Several downstream components involved in the stress response are controlled by DAF-2/insulin/IGF-signaling pathway in *C. elegans*. Reduced *daf-2* leads to the activation of a transcription factor DAF-16/FOXO, a key regulator of a wide range of genes involved in antioxidant activities (e.g., catalase and Mn-superoxide dismutase), chaperones (*hsps*) as well as antimicrobial and metabolic genes involved in stress resistance [25]. *daf-16* activity is essential for stress response in *C. elegans* [26,27]. Under normal growth conditions, *daf-16* is usually restricted primarily in the cytosol region, however, it rapidly translocates to the nucleus under heat stress [12] and this pathway was an underlying mechanism in the increased thermo-tolerance in TWE-treated *C. elegans*. However, both the control and TWE-treated worms showed *daf-16* translocation to the nuclei under heat-shocked condition suggesting that the *daf-16* was induced with heat stress. But *daf16* transcript expression was higher in TWE-treated worms which indicate that *daf16* was activated more following TWE treatment. However, the higher survival of *daf-16* mutant under thermal stress confirmed that the observed beneficial effects of TWE involved mechanisms independent of *daf-16* activity. Hsu *et al.* [28] reported that the genes coding small heat-shock proteins are targets of *daf-16* as a protective mechanism. Libina *et al.* [29] demonstrated that the daf-16 regulates longevity primarily from intestinal cells which can be regulated through cell-autonomous and non-autonomous pathways. It has been suggested that the heat-shock transcription factor may be involved in regulating the lifespan in association with *daf-16* [30]. It has been observed that during thermal stress, heat shock response is induced in organisms which is controlled by the heat shock factor–1 (HSF-1), that triggers the cytoprotective heat shock proteins (HSPs) regulating aging by sustaining longevity, and has also been implicated in age-associated neuro-degeneration [31,32]. These HSPs, act as molecular chaperones that are important in stress resistance. Our results showed an up regulation of *hsp16.2* transcript under heat-stress regardless of the treatments. Nevertheless, the expression level was much higher in TWE treated worms indicating that HSPs may have some role to play in TWE induced heat stress tolerance. Prahlad *et al.* [19] suggested that thermosensory neuron, AFD, rather than cell autonomy is involved in the heat shock response of *C. elegans*, leading to the integration of behavioral, metabolic, and stress-related responses into an organismal response against thermal stress. This implies that defense mechanisms other than chaperones, contributed in *C. elegans* tolerance to heat stress. It has been suggested that other redundant mechanisms do play pivotal roles in survival of the organism under stress conditions [33] and we suggest that one such mechanism could be the antioxidant potential of the TWE.

Environmental stresses (e.g., thermal stress) have been shown to cause an increase in reactive oxygen species (ROS) accumulation in *C. elegans* [34]. Several compounds such as ginkgolipids, flavonoids attenuate ROS production in the nematode. It was observed that the ROS level was much lower in TWE-treated nematodes and similar results were observed in vitro with a strong DPPH scavenging activity and Fe^2+^ chelating ability of the extract (data not shown). Interestingly, the expression of *sod-3::gfp* transgenic line was reduced by TWE treatment in *C. elegans* but the transcript level increased. Perhaps these activities are differently controlled in the organisms. Thus, it could be assumed that the observed beneficial effects of TWE treatment in *C. elegans* are mediated, at least in part, by direct antioxidative properties. Similar mechanism was observed with antioxidants, such as quercetin [34] and Ginkgo biloba extract EGB 761 [35]. Hence, it may be speculated that in the presence of the TWE, the cellular oxidative status of *C. elegans* was reduced, and that led to less radicals generated in the nematodes.

Both *sod3* and *hsp* play important roles in stress tolerance [36] and in the present study, the TWE-treated nematodes had higher induction of these genes under thermal stress. Both *sod-3* and *hsp-16.2* are down-stream effectors of *daf-16* that acts as stress-response reporters in *C. elegans* [37]. Up-regulation of these genes in TWE-treated *C. elegans* without a significant effect on *daf-16* suggested that TWE exerted thermal tolerance via a novel *daf-16* independent pathway. Interestingly, higher transcripts of *sod-3* and *hsp-16.2* in *C. elegans* exposed to TWE treatments did not correlate with lower *sod-3::gfp* and *hsp-16::gfp* expression in the transgenic nematodes with marker protein fused with these stress-related genes. One factor that might have contributed to this difference could be that the samples for GFP analysis were taken several hours after the nematodes were placed in stress recovery phase at 20 ºC which might have altered the expression pattern of the reporter genes. Previous studies have also demonstrated that nematodes expressing high *gfp* levels in the *hsp::gfp16* strain TJ375, after heat shock (35 °C), were more thermo-tolerant than nematodes with low GFP.

This study further revealed the effect of TWE in *C. elegans* proteome maps under heat stress. The functions of identified differential proteins indicated the importance of such proteins in thermo-protection. The results presented here showed that approximately 60% of the identified proteins were related to general and energy metabolism, protein binding, protein synthesis and electron transport. Similar to previous reports [19,38], all these proteins are known to be directly involved in stress tolerance in *C. elegans.*

This study identified high concentration of several cytoskeletal proteins in TWE treatment that could play a role in stabilizing metabolic activity under heat stress. These proteins were not expressed in the control worm exposed to heat stress. Cytoskeletal proteins such as actin and myosin are responsible for movements and cellular locations and are likely to interact with mitochondria [39]. The results showed several members of the mitochondrial carrier protein family such as ATP/ADP exchange, uptake of phosphate, glutamate and folate, as well as the transport of ions, lipids, nucleotides were highly expressed in TWE treatment. Excess production of ROS may occur due to defects in electron transport complexes or other disturbances to the mitochondria and the increase in ROS is associated with a variety of pathologies and aging. In this study we observed TWE treatment up regulate a number of proteins that affect cell redox homeostasis, mainly involved in antioxidant defense mechanism against ROS, such as H_2_O_2_, OH^−^, and O^2−^. The intra-mitochondrial signaling and the communication of mitochondria with the cytoplasm and other organelles are very important [40]. Some of the proteins, identified in the present study include several peroxiredoxin protein 2, ATP synthase sub-unit beta, Protein K08E3.4, cytochrome c oxidase sub-unit 5, and carbamoyl-phosphate synthase L chain ATP-binding protein which are involved in recognition and communication of mitochondrial signaling.

Proteins classified under other functional classes are also known to play a role in signal transduction. For example, proteins such as the multi-functional cytochrome c and the mitochondrial channel proteins, are also known to be involved in the calcium signaling pathway. The antioxidant proteins may also take part in regulation, such as Oxidative phosphorylation, cellular redox signaling and mitochondrial gene expression [11]. A number of differentially regulated proteins identified in this study grouped in ATP synthesis, amino acid metabolism, biosynthesis and organic acid metabolism. General metabolic process, energy metabolism, nucleotide/protein binding, electron transport, protein synthesis, proteolysis, response to heat, stress response and signal transduction presented as pie chart (Figure 11). Most of them have potent roles in energy metabolic process and the well studied mitochondrial metabolic pathway. These results are in agreement with the central role of mitochondria in the cellular metabolic processes. In addition to the obvious predominance of proteins in the general and energy metabolism, the other two largest groups of specific proteins were identified to be associated with heat stress and signal transduction. About 25% of the metabolism-related and 13% of growth-related proteins in this study are attributable to functions such as mitochondrial protein biosynthesis and translocation, respectively. Prohibitins, kinases and other factors regulating mitochondrial biogenesis identified in this study have also been implicated in biological processes.

Seaweeds are known to have a wealth of bioactive compounds including alginates, laminarins, fucans and fucoidan [3,5]. It has been recognized that polysaccharides display physiological and biological activities including immuno-modulatory and antioxidant activity [41].

In conclusion, we suggest that the thermo-tolerance activity of TWE is probably due to the presence of diverse polysaccharides in the extract. However, further studies are being conducted to pinpoint the compound(s) responsible for such effects. In this study, the life span enhancing response of TWE in the nematode *C. elegans* under heat stress in part may be ascribed to its direct ROS scavenging activity as well as by the up-regulation of stress tolerance related genes such as *sod-3* and *hsp-16.2* and proteins involved in stress responses. Taken together, our results indicated that the compound present in TWE may play a role in extending the life expectancy of the nematode by offering a protection against heat stress. Further research is needed to find out the nature of these compounds and what role they play in stress tolerance mechanism.

## 4. Experimental Section

### 4.1. Chemicals and *C. elegans* Strains

All chemicals were purchased from Sigma Aldrich, Oakville, Ontario, Canada, unless otherwise stated. Samples of Tasco^®^ powder were supplied by Acadian Seaplants Limited, Dartmouth, Nova Scotia, Canada. The wild type *Caenorhabditis elegans* strain N2 (var. Bristol), the transgenic strains CF1553 [muIs84[pAD76(*sod-3::gfp*)], CL2070 [dvIs70(*hsp-16.2::gfp*; rol-6(su1006))], CL2166 [dvIs19(pAF15(*gst-4::gfp*::NLS))], GR1307 [*daf-16*(mgDf50)], and TJ356 [*zIs3*56 [*daf-16::gfp* rol-6(su1006)], and *Escherichia coli* strain OP50 were obtained from the *Caenorhabditis* Genetics Center, University of Minnesota, USA. Cultures of OP50 *E. coli* were grown overnight in LB broth and concentrated 10 times by centrifugation at 3500 × g for 10 min. The *C. elegans* strains were maintained at 20 °C on 1.2% solid nematode growth medium (NGM) [42] seeded with 50 μL of live OP50 *E. coli* as a food source.

### 4.2. Preparation of Tasco^®^ Water Extract (TWE)

Aqueous extract of Tasco^®^ (TWE) was prepared by dissolving 10 g of the product in 40 mL distilled water at 70 °C for 1 h. The mixture was centrifuged at 10,000 × g for 10 min at room temperature and the supernatant was pipetted out and put in a new tube. The pellet was redissolved in 40 mL water and the extraction procedure was repeated. The resulting supernatants were combined, dried under nitrogen (N_2_) gas and stored at 4 °C until use. A stock solution equivalent to −40 mg/mL of Tasco^®^ was prepared in distilled water.

### 4.3. Thermal-Tolerance Assay

Synchronized N2 eggs were placed on NGM plates (~25 eggs/plate) containing TWE at various concentrations (*i.e.*, 20, 100, 300, 600 or 1000 μg/mL) or vehicle (distilled water) with heat-killed OP50 as food source and maintained at 20 °C. Three day old adult worms, treated with TWE, were exposed to two thermal stress temperatures *i.e.*, 30 °C and 35 °C in an incubator (Innova, New Brunswick, Canada). At 30 °C, the mortality of the worms was enumerated at 24 h intervals whereas at 35 °C, the number of individuals was counted every 2 h until all the worms had died.

### 4.4. Stress Response Assay with Mutants and Transgenic Strains of *C. elegans*

To study the involvement of daf-16, a FOXO-family transcription factor that maneuvers the rate of ageing of *C. elegans*, in TWE-mediated stress tolerance, young adults of *daf-16* loss-of-function mutant (GR1307) and wild type (N2) nematodes were incubated at 35 °C for 7 and 9 h, respectively, and the number of surviving individuals were recorded. *C. elegans* strains CL2070, CF1553 and CL2166 containing inducible GFP reporter for *hsp-16.2*, *sod-3* and *gst-4*, respectively were cultivated on treatment plates from eggs for 3 days. For strains CF1553 and CF2166 the photographs were taken directly at day 3, whereas for strain CL2070, the worms were exposed to 35 °C for 2 h and allowed to recover at 20 °C for 4 h before photography. A total of 90 nematodes were used for each strain per condition. For *daf-16::gfp* localization, TJ356 *C. elegans* were used. Nematodes from normal or the heat stress conditions (*i.e.*, 35°C for 2 h, followed by recovery at 20 °C for 4 h) were placed on 2% agar plates and pictures were taken using a fluorescence microscope. Epi-fluorescence images were acquired at constant exposure using the 20× objective lens. Fluorescence images were acquired at identical exposure times using 20× magnification for each strain. The fluorescence intensity for the head part of the worms was measured using the free Java image processing program ImageJ [43]

### 4.5. Life Span Assay

Synchronized *C. elegans* N2 strain eggs were placed on NGM plates (~25 eggs/plate) containing TWE or “vehicle” (distilled water) and maintained at 20 °C. The experimental plates were prepared by adding various concentrations (*i.e.*, 20, 100, 300, 600, or 1000 μg/mL) of TWE stock solution to NGM (at 55 °C) having heat-killed the *E. coli* OP50 (at 65 °C for 30 min) to be used as food for the nematode. After 3 days, young adult worms were transferred to fresh treatment plates supplemented with 200 μM 5-fluorodeoxyuridine (FUdR) which was used to prevent development of progeny [44]. The nematodes were transferred to the new treatment plates every 2 days. Survival was evaluated daily and the animals were scored as dead if they failed to respond to gentle, repeated touches with a platinum pick. The first day of adulthood was considered as day 1. Individuals that crawled off the walls of the plates and died from desiccation were excluded from the analysis.

### 4.6. Body Size Analysis

Synchronized *C. elegans* eggs were cultured on treatment plates at 20 °C for 6 days. Forty five individuals per treatment were mounted on 2% agar pads (in M9 buffer) and paralyzed with 25 mM sodium azide (in M9 buffer) and photographed under a fluorescence microscope. Length and width of the individuals were measured using ImageJ (NIH).

### 4.7. Brood Size Assay

Synchronous eggs of the N2 strain were obtained by allowing the adults to lay eggs for 1 h and incubated at 20 °C on for 2 days. The larvae at L4 stage (30 per treatment) were transferred individually to treatment plates for egg laying. The worms were transferred to fresh plates daily until reproduction was completed. The mean number of offspring for each worm was counted beginning 48 h after egg-laying.

### 4.8. Feeding Rate Assay

Synchronized eggs (N2 strain) were placed on treatment plates spotted with heat-killed OP50 *E. coli*, raised at 20 °C, and transferred to fresh plates with equal food/TWE treatments every 2 days after reaching adulthood. Thirty nematodes were selected randomly for each treatment and their feeding rate was scored by counting their pharyngeal bulb contractions over a 20 s period, using a microscope at room temperature (22 °C) (see [45]). The pharyngeal pumping rate was counted every 2 days for the first 9 days of adulthood.

### 4.9. Measurement of Reactive Oxygen Species (ROS)

Intracellular ROS in *C. elegans* were measured with H_2_DCF-DA as the molecular probe. For ROS detection without any stress conditions, the L4 worms from seaweed treated or untreated conditions were used. The wild type N2 worms were treated the ROS detection under oxidative stress. The L4 worms from TWE 300 μg/mL treated plates along with untreated plates were incubated along with 300 μM juglone in the microplate reader during fluorescence detection. Briefly, the *C. elegans* were collected into 100 μL phosphate-buffered saline (PBS) with 1% Tween 20 in centrifuge tubes and pipetted into the black flat-bottom 96-wellmicrotiter plate (Costar) containing H_2_DCF-DA (final concentration 50 μM in PBS). Samples were read every 2 h for 6 h in a Biotek microplate reader at 37 °C with excitation 485 nm and emission 530 nm.

### 4.10. Expression Analysis of Stress Induced Genes in *C. elegans*

*C. elegans* strain N2 was grown and maintained at 20 °C as described above. The N2 adults were allowed to lay eggs on NGM plates for 2 h at 20 °C. The eggs were transferred to control or TWE-supplemented NGM plates until the young adult stage was developed. The worms were heat-shocked (35 °C) for 2 h and 80–100 worms were picked directly into TRIzol Reagent (Invitrogen Life Technologies). The samples were flash frozen in liquid nitrogen. Total RNA was extracted based on the TRIzol method (Invitrogen) with minor modifications. Briefly, 100 μL of TRIzol with worms was added to 70 μL chloroform, mixed well and centrifuged at 10,000 × g for 10 min. The supernatant was mixed with 70 μL ethanol (70%) and loaded directly onto RNeasy spin columns (Qiagen, Canada) to precipitate RNA according to the manufacturer’s protocol. The quality and quantity of RNA obtained was assessed with Nanodrop ND-1000 (NanoDrop Technologies Wilmington, DE) and formaldehyde gel electrophoresis. Total RNA was reverse transcribed with 500 μg of DNA-ase digested total-RNA using Quantiscript reverse transcriptase (Qiagen, Canada) following the manufacturer’s instructions.

The induction of four genes: viz: *hsp-16.2*, *daf-16*, *sod-3* and *skn-1* was used to determine the heat stress response of *C. elegans* treated with TWE. The *daf-16*, *sod-3*, *skn-1* and *hsp-16.2* are known to be important in the life-span and stress tolerance in *C. elegans*. The primers used for qReal Time PCR were as follows: *hsp-16.2*, forward 5′-ACGCCAATTTGCTCCAGTCT-3′, Reverse 5′-GATGGCAAACTTTTGATCATTGTTA-3′; *daf-16*, forward 5′-TTTCCGTCCCCGAACTCAA-3′, reverse 5′-TTCGCCAACCCATGATGG-3′; *sod-3*, forward 5′-AGCATCATGCCACCTACGTGA-3′, reverse 5′-CACCACCATTGAATTTCAGCG-3′; *skn-1*, forward 5′-AGTGTCGGCGTTCCAGATTTC-3′ and reverse 5′-GTCGACGAATCTTGCGAATCA-3′; *ama-1*, forward 5′-CTGACCCAAAGAACA CGGTGA-3′, reverse 5′-TCCAATTCGATCCGAAGAAGC-3′. The *C. elegans* gene *ama-1* was used as an internal control for quantification. Quantitative, Real-Time PCR was performed on StepOne™ Real-Time PCR System (Applied Biosystems) following the manufacturer’s instructions using SYBR green reagent (Roche Diagnostics, Mississauga, ON, Canada). Data were analyzed from two independent runs.

### 4.11. Proteome Analysis of TWE Treated *C. elegans*

#### 4.11.1. Preparation of Synchronized Nematode Populations

In order to produce a synchronized population, young adult nematodes were transferred to the NGM plates. The eggs were transferred to TWE (300 μg/mL) or control plates containing heat-killed *E. coli* OP 50 as food source. The nematodes were allowed to develop for 3 days on treatment plates and young adults were drained in to eppendorf tubes using 30% sucrose as the floating solution. The individuals were washed with 0.5 M TBS buffer several times to remove contamination by *E. coli*. After several washes, the pure nematode population was used for protein extraction.

#### 4.11.2. Treatment Conditions

Four treatment conditions were analyzed for the *C. elegans* proteome. Briefly, plain (NGM only) or TWE supplemented NGM in Petri plates were prepared, placed with synchronized nematode eggs and each treatment was subjected to 20 and 35 °C for proteome analysis. The worms were collected separately from all treatments and purified for protein isolation.

#### 4.11.3. Protein Extraction

Frozen worms were ground in a mortar using liquid nitrogen and suspended in 10% trichloracetic acid in acetone with 0.07% dithiothreitol (DTT) and kept at 20 °C for 1 h, followed by centrifugation for 15 min at 35,000 × g. The pellets were washed once with ice cold acetone containing 0.07% DTT at −20 °C for 1 h and centrifuged again for 15 min at 35,000 × g. Washing was repeated four to five times until the supernatant was clear. The final precipitated pellet was lyophilized for 2 h. About 10 mg of dried powder was used for protein extraction by dissolving in 350 μL of sample lysis buffer containing 7 M urea, 2 M thiourea, 4% 3-[(3-cholamidopropyl)dimethylammonio]-1-propanesulfonate, 0.5% ampholytes (GE Healthcare, Uppsala, Sweden) and 0.7% DTT. Protein extraction was carried out at 37 °C with occasional vortexing. After 1 hr incubation, tissues were pelleted by centrifuging for 30 min at 35,000 g at room temperature. The supernatant was distributed in 100 μL aliquots and kept at −80 °C before 2D-PAGE analysis [46]. Protein content was determined by the Bradford Method [47].

#### 4.11.4. 2D SDS-PAGE

Equal amounts of protein (100 μg) from the control and TWE-treated samples were separated by 2D-PAGE. In the first dimension, 17 cm IPG strips and pH 4–7 were used. Electrophoresis was conducted at 500 V for 1 h, followed by 1000 V for 1 h and 1750 for 24 h. After IEF, the proteins were separated by SDS–PAGE in the second dimension using 12% polyacrylamide gels [48]. The gels were silver stained following the protocol of Shevchenko *et al.* [49]. For each replicate, one set of gels with high resolution, running at different times, were selected for further analysis. The relative abundance of protein spots was quantified with PD Quest software (Biorad, Hercules, CA) after silver staining the gels and scanning with a GS-700 imaging densitometer (Bio-Rad, Hercules, CA).

### 4.12. Protein Identification

#### 4.12.1. Protein Digestion

Differential protein spots were excised from the preparative gels. The excised protein spots were digested with trypsin using the MassPREP station (Waters Milford, MA, USA). The excised spots were de-stained with 50 μL of 50 mM ammonium bicarbonate and 50 μL of 50% acetonitrile, washed once with 50 μL of 100 mM ammonium bicarbonate and 50 μL of dehydrated acetonitrile. Digestion was made with 6 ng μL^−1^ trypsin in 25 μL of 50 mM ammonium bicarbonate for 5 h at 37 °C. The digested protein was extracted twice [first with 1% formic acid (30 μL), and second with 1% formic acid (12 μL)/50% acetonitrile (12 μL)]. The digested proteins were combined and maintained on a PCR plate at 4 °C for further analysis.

#### 4.12.2. Protein Identification and Sequencing by 2D Nano LC-MS/MS

Protein identification and sequencing was carried out using two-dimensional liquid chromatography ESI-MS (Agilent 1100 series 2DnanoLC-MS). Tryptic digested protein was subjected to chromatography column followed by reverse phase separation. Peptides were ionized in the liquid phase in the electro-spray ionizer before entering the ion trap, where they were fragmented (MS/MS) and detected. The data were sent to the MASCOT search engine (Agilent) for analysis.

#### 4.12.3. Database Searching with MS/MS Spectra

MS/MS spectra were used to search against the NCBI non-redundant protein database using MS/MS Ion Search Engine, and then used to conduct protein identifications based on matching MS/MS spectra of a protein with a protein or DNA sequence data base [50]. The significance of the protein match with the ion score was based on the “Mouse” scoring algorithm. The ion score was calculated as −10 × LOG10(P), where P was the absolute probability that the observed match was a random event. Thus, a relatively small P value meant that the match of identified protein and the MS/MS spectra was not a random event. A significant specific match increased the ion core, so a high score meant highly significant matching (MASCOT Help) [51]. A single protein, having a higher score than the minimum score for the significance level (*P* < 0.05), was judged as a significant match. In each MASCOT search output result, the minimum score for the significance level was provided, based on the absolute probability and size of the sequence database being searched.

### 4.13. Statistical Analysis

Each experiment was replicated at least three times with independent trials. Data were represented as mean ± SEM (standard error of the mean) and were analyzed by ANOVA and differences between groups were considered statistically significant at *P* < 0.05 using Tukeys HSD test with COSTAT^®^ statistical software. Results from the life-span experiment were processed using the Kaplan-Meier survival analysis of SPSS 15.0 and compared amongst the control and the TWE treated groups for significance by means of a log-rank, pairwise comparison test.

## 5. Conclusions

Water soluble components of Tasco^®^, elicited heat stress tolerance in *C. elegans*. The induced stress tolerance was associated with differential regulation of a number of stress response genes including *sod3* and *hsp16-2*. The proteome analysis revealed significant differences in Tasco^®^ treated *C. elegans* leading to protection against high temperature stress. Taken together, our results suggest that the *A. nodosum* product alters physiology of *C. elegans* making it more resistant to heat stress.

## Figures and Tables

**Table 1:** Effects of water extract of Tasco^®^ on longevity of *C. elegans* N2 when started from eggs (SE = standard error).

Treatment (20 °C)	Total (N)	Censored (N)	Adult Life Span, Days (Mean ± SE)	*P vs.* Control (log-Rank)
Control	125	0	20.56 ± 0.47	
TWE 20 μg/mL	115	0	22.09 ± 0.52	0.055
TWE 100 μg/mL	185	0	23.24 ± 0.47	0.0003
TWE 300 μg/mL	118	0	24.02 ± 0.72	0.00004
TWE 600 μg/mL	145	0	20.85 ± 0.39	0.899
TWE 1000 μg/mL	140	0	21.66 ± 0.37	0.175

**Table 2:** Proteins that were more abundant/differentially (up/down) expressed in TWE-treated *C. elegans* under 35 °C stress.

ID Number a	Name of Protein	Increase Fold in Treated Larva b	Accession Number	Molecular Mass (Da)/pI	Coverage (%)	Matched Peaks	Putative Functions
	**Up regulated proteins following TWE treatment**						
1	O62040_CAEEL Putative uncharacterized protein OS = *Caenorhabditis elegans*	D	O62040	37,708	2.37%	1	Strictosidine synthase (a key enzyme in alkaloid biosynthesis) activity
2	Q9N5D3_CAEEL Drosophila sos homolog protein 1 OS = *Caenorhabditis elegans* GN = SOS-1	D	Q9N5D3	169,902	0.47%	1	DNA binding, Rho guanyl-nucleotide exchange factor activity, calmodulin binding
3	AL7A1_CAEEL Putative aldehyde dehydrogenase family 7 member A1 homolog OS = *Caenorhabditis elegans*	D	P46562	56,994.8	4.52%	3	Detoxification of aldehydes generated by alcohol metabolism and lipid peroxidation, Protects cells from oxidative stress.
4	HSP17_CAEEL Heat shock protein HSP-16.48/HSP-16.49 OS = *Caenorhabditis elegans* GN	D	P02513	16,282	4.9%	1	Determination of adult lifespan, endoplasmic reticulum unfolded protein response, response to heat
5	RSSA_CAEEL 40S ribosomal protein SA OS = *Caenorhabditis elegansCaenorhabditis elegans* GN = RPS-0 PE = 1 SV = 3	D	P46769	30,685.1	6.16%	2	Assembly and/or stability of the 40S ribosomal subunit. and processing of the 20S rRNA-precursor to mature 18S rRNA in a late step of the maturation of 40S ribosomal subunits
6	Q9XW04_CAEEL Protein Y18D10A.21, partially confirmed by transcript evidence OS = C	D	Q9XW04	32,897.3	2.8%	1	Catalytic activity, cation binding, carbohydrate metabolic process
7	Q94255_CAEEL Saposin-like protein family protein 20	D	Q94255	24,484.9	2.76%	1	Lipid metabolism
8	Q17849_CAEEL Heat shock protein protein 25, isoform a OS=*Caenorhabditis elegans*	D	Q17849	25,238.6	4.57%	1	Response to heat, protein binding, Stress response
9	D0LGS3_HALO1 Carbamoyl-phosphate synthase L chain ATP-binding protein OS = Haliang	1.89	D0LGS3	211,022	0.47%	1	ATP binding, biotin binding, ligase activity
10	C3JZ86_PSEFS Superoxide dismutase OS = Pseudomonas fluorescens (strain SBW25) GN = P	D	A0KJL0	22,743.6	4.57%	1	superoxide dismutase activity, oxidation reduction, superoxide metabolic process
11	Q18100_CAEEL Sensory axon guidance protein 7	D	Q18100	127,605	0.61%	1	Involved in specific pathways by attractive and repulsive cues in the extracellular environment
12	Q6EUT7_CAEEL Protein ZK1151.1g, partially confirmed by transcript evidence OS = *Caenorhabditis elegans*	1.88	Q6EUT7	561,703	0.2%	1	Cell cycle arrest, Actin binding and Calcium ion binding
13	14331_CAEEL 14-3-3-like protein 1 OS = *Caenorhabditis elegans* GN = PAR-5 PE = 1 SV = 2|6	1.52	P41932	28,173	10.1%	3	Adapter protein involved in regulation of general and specialized signaling pathway
14	C5IWV5_PIG Trypsinogen OS = Sus scrofa PE = 2 SV = 1	1.57	C5IWV5	24,391.3	7.79%	2	Proteolysis, serine-type endopeptidase activity
15	MLR1_CAEEL Myosin regulatory light chain 1 OS = *Caenorhabditis elegans* GN = MLC-1 PE	4.9	P19625	8586.4	31.2%	15	Calcium ion binding, motor activity
16	MLR1_CAEEL Myosin regulatory light chain 1 OS = *Caenorhabditis elegans* GN = MLC-1 PE	D	P19625	8586.4	31.2%	6	Increases myosin filament stability
17	C5IWV5_PIG Trypsinogen OS = Sus scrofa PE = 2 SV = 1	D	C5IWV5	24,391.3	7.79%	2	Proteolysis, serine-type endopeptidase activity
18	HSP11_CAEEL Heat shock protein HSP-16.1/HSP-16.11 OS = *Caenorhabditis elegans* GN = h	D	P34696	16,235.5	18.6%	1	Defense response, determination of adult lifespan, positive regulation of growth rate, response to heat
19	COX5A_CAEEL Cytochrome c oxidase subunit 5A, mitochondrial OS = *Caenorhabditis elegans*	4.31	P55954	20,093.3	5.17%	1	Cytochrome-c oxidase activity, metal ion binding
20	O45509_CAEEL Protein F41D3.10, partially confirmed by transcript evidence OS = *Caenorhabditis elegans*	D	O45509	76,674	1.19%	1	Sequence-specific DNA binding, steroid hormone receptor activity, zinc ion binding
21	Q9UAX1_CAEEL Putative uncharacterized protein T12B3.4 OS = *Caenorhabditis elegans*	D	Q9UAX1	33,698.7	2.74%	2	Protein binding
22	HSP17_CAEEL Heat shock protein HSP-16.48/HSP-16.49 OS = *Caenorhabditis elegans* GN	D	P02513	16,282	11.2%	5	Determination of adult lifespan, endoplasmic reticulum unfolded protein response, response to heat
23	HSP11_CAEEL Heat shock protein HSP-16.1/HSP-16.11 OS = *Caenorhabditis elegans* GN = h	1.2	P34696	16,235.5	33.8%	6	Defense response, determination of adult lifespan, response to heat
24	O18180_CAEEL Protein W09D10.3, confirmed by transcript evidence OS = *Caenorhabditis elegans*	2.36	O18180	18,421.4	16.8%	5	Determination of adult lifespan, positive regulation of growth rate, structural constituent of ribosome
25	O44751_CAEEL Putative uncharacterized protein OS = *Caenorhabditis elegans*	2.02	O44751	55,504.4	1.23%	1	ATP binding, protein kinase activity
26	Q9XUT0_CAEEL Protein K08E3.4, confirmed by transcript evidence OS = *Caenorhabditis elegans*	11.14	Q9XUT0	71,922.8	3.11%	2	Actin binding
27	O45177_CAEEL Putative uncharacterized protein OS = *Caenorhabditis elegans* GN = K07H8	10.63	O45177	40,387	2.31%	1	RNA binding
28	C5IWV5_PIG Trypsinogen OS = Sus scrofa PE = 2 SV = 1	1.0	C3JZ86	21,959.7	4.55%	1	Proteolysis, serine-type endopeptidase activity
29	ATPB_CAEEL ATP synthase subunit beta, mitochondrial OS = *Caenorhabditis elegans* GN	1.59	P46561	57,509.3	1.86%	1	ATP binding, hydrogen ion transporting ATP synthase activity
30	GPX2_CAEEL Probable glutathione peroxidase R05H10.5 OS = *Caenorhabditis elegans* GN	3.91	O62327	18,134.3	18.4%	3	GSH-Px -an antioxidant, Important role in the metabolism of certain hydroperoxides
31	Q21057_CAEEL Galectin OS = *Caenorhabditis elegans* PE = 2 SV = 1|6239	3.79	Q21057	15,911.4	26%	4	Sugar binding
32	Q20804_CAEEL CNB-1 OS = *Caenorhabditis elegans* GN = CNB-1 PE = 2 SV = 3|6239	1.01	Q20804	19,654.9	5.85%	1	Calcium ion binding
33	14331_CAEEL 14-3-3-like protein 1 OS = *Caenorhabditis elegans* GN = PAR-5 PE = 1 SV = 2|6	D	P41932	28,173	7.26%	2	Protein domain specific binding, dauer entry, determination of adult lifespan
34	HSP17_CAEEL Heat shock protein HSP-16.48/HSP-16.49 OS = *Caenorhabditis elegans* GN	8.43	P02513	16,282	11.2%	2	Determination of adult lifespan, endoplasmic reticulum unfolded protein response to heat
	**Down regulated proteins due to treatment**						
35	IF5A1_CAEEL Eukaryotic translation initiation factor 5A-1 OS = Caenorhabditis eleg	D	P34563	17,849.3	6.83%	2	RNA binding, ribosome binding, translation elongation factor activity
36	Q20644_CAEEL Protein F52B5.3, partially confirmed by transcript evidence OS = *Caenorhabditis elegans*	D	Q20644	162,454	0.7%	1	Inorganic anion exchanger activity
37	EIF3C_CAEEL Eukaryotic translation initiation factor 3 subunit C OS = *Caenorhabditis elegans*	D	O02328	103,827	0.67%	1	Protein synthesis, translation initiation factor activity
38	RL18_CAEEL 60S ribosomal protein L18 OS = *Caenorhabditis elegans* GN = RPL-18 PE = 3 SV	0.30	O45946	51,385.2	3.72%	1	RNA binding, Structural constituent of ribosome
39	Q23440_CAEEL Protein ZK1307.8, confirmed by transcript evidence OS = *Caenorhabditis elegans*	0.46	Q23440	58,045.8	1.38%	1	Calcium ion binding, protein binding
40	A8DYR6_CAEEL Peroxiredoxin protein 2, isoform b OS = *Caenorhabditis elegans* GN	0.80	A8DYR6	21,767	14.9%	3	Thioredoxin peroxidase activity, determination of adult lifespan, hydrogen peroxide catabolic process

D The protein was found to be observed in the TWE treated gels (Stressed/unstressed);

a ID number indicates the protein spot in the 2DE master reference gel;

b The mean (*n* = 3) factor of increase/decrease in spot due to treatment compared to control worms, obtained from the three different gels
